# Comparative transcriptomic and proteomic analyses of the green and white parts of chimeric leaves in *Ananas comosus* var. *bracteatus*

**DOI:** 10.7717/peerj.7261

**Published:** 2019-07-10

**Authors:** Yanbin Xue, Jun Ma, Yehua He, Sanmiao Yu, Zhen Lin, Yingyuan Xiong, Fatima Rafique, Fuxing Jiang, Lingxia Sun, Mingdong Ma, Yujue Zhou, Xi Li, Zhuo Huang

**Affiliations:** 1 College of Landscape Architecture, Sichuan Agricultural University, Chengdu, Sichuan, China; 2 Horticultural Biotechnology College of South China Agricultural University, South China Agricultural University, Guangzhou, Guangdong, China

**Keywords:** Molecular mechanism, Chimeric leaves, *Ananas comosus* var. *bracteatus*, Transcriptome, Proteome

## Abstract

**Background:**

*Ananas comosus* var. *bracteatus* has high ornamental value due to its chimeric leaves. However, the chimeric trait is very unstable in red pineapple plants, and transcriptional variation between the two types of cells (white/green cells) and the molecular mechanism responsible for their albino phenotype remain poorly understood.

**Methods:**

Comparative transcriptomic and proteomic analyses of the white parts (Whs) and green parts (Grs) of chimeric leaves were performed.

**Results:**

In total, 1,685 differentially expressed genes (DEGs) (712 upregulated and 973 downregulated) and 1,813 differentially abundant proteins (DAPs) (1,018 with low abundance and 795 with high abundance) were identified. Based on Gene Ontology (Go) and Kyoto Encyclopedia of Genes and Genomes (KEGG) pathway enrichment analyses, the DEGs were mostly involved in carbon fixation in photosynthetic organisms, porphyrin and chlorophyll metabolism and oxidative phosphorylation, while proteomic analysis revealed that DAPs were mostly related to ribosomes, photosynthesis, photosynthesis antennas, and porphyrin and chlorophyll metabolism. Combined analysis showed increased mRNA levels but low abundance of nine proteins level in Whs /Grs related to photosynthetic pigment and photosynthesis. Transcriptional changes, posttranscriptional regulation and translational alterations of key enzymes involved in chlorophyll biosynthesis and photosynthesis may play important roles in the albino parts of chimeric leaves.

## Introduction

*Ananas comosus* var. *bracteatus* is an herbaceous perennial monocot that belongs to the Bromeliaceae family (Collins, 1968). Plants of this family are mostly cultivated commercially for their fruit, the high-quality silk fiber of their stem and their chimeric leaves. Indeed, *A. comosus* var. *bracteatus* has high ornamental value due to its green/white chimeric leaves and decorative red fruits and is used as an indoor potted plant, event decoration and cut flower material. Leaf cross-sections have revealed that the chimeric leaves of this plant are composed of normal green cells and albino white cells. The red pineapple plant is self-incompatible and is predominantly propagated by division for production, though the plants regenerated via this propagation method are low in quality compared to those obtained by tissue culture. In fact, tissue culture is very convenient and effective method of cultivating red pineapple plant, yet the chimeric trait is very unstable, and only 1% of the plants regenerated by tissue culture are chimeric (Cao, 2011). Therefore, study of the mechanism by which chimeric traits occur in *A*. *comosus* var. *bracteatus* is of great theoretical and practical importance for enhancing the stability of the chimeric character. Leaf color mutants are excellent material for investigations of the chlorophyll metabolic pathway, chloroplast development, and photosynthesis (Larkin et al., 2003; Wang et al., 2012). Nonetheless, the transcriptional variation between normal and albino cells and the molecular mechanism responsible for the albino trait remain poorly understood.

With regard to investigations of the genetic diversity of the genus *Ananas*, we previously performed transcriptome sequencing of the leaves, stems and roots of *A*. *comosus* var. *bracteatus* (Ma et al., 2015), which provided a basic transcriptomic database for this species. Furthermore, comparative transcriptomic analyses of completely white (CWh) and completely green (CGr) leaves of *A*. *comosus* var. *bracteatus* shoots have revealed that transcriptional differences play vital roles in the biosynthesis of photosynthetic pigments and in the development of chloroplasts, which might cause differences in photosynthesis and leaf color (Li et al., 2017). miRNAs involved in the development of CGr and CWh leaves have been identified and the regulatory functions of miRNAs in albino leaf cells examined (Xiong et al., 2018).

In the present study, a transcriptomic data set generated from Whs/Grs of the chimeric leaves** of *A*. *comosus* var. *bracteatus* was first obtained via RNA-Seq. We then examined the expression patterns of genes at the transcriptomic and proteomic levels using the Tandem Mass Tag (TMT) quantitative strategy and LC-MS/MS analysis (Guo et al., 2017). We performed functional analysis of DEGs/DAPs and assessed Gene Ontology (GO) annotations and Kyoto Encyclopedia of Genes and Genomes (KEGG) pathway enrichment analysis. Overall, our integrated analysis allows for a better understanding of the molecular changes that occur within the chimeric leaves of *A. comosus* var. *bracteatus*.

## Materials & Methods

### Plant materials and sample preparation

**Figure 1:**
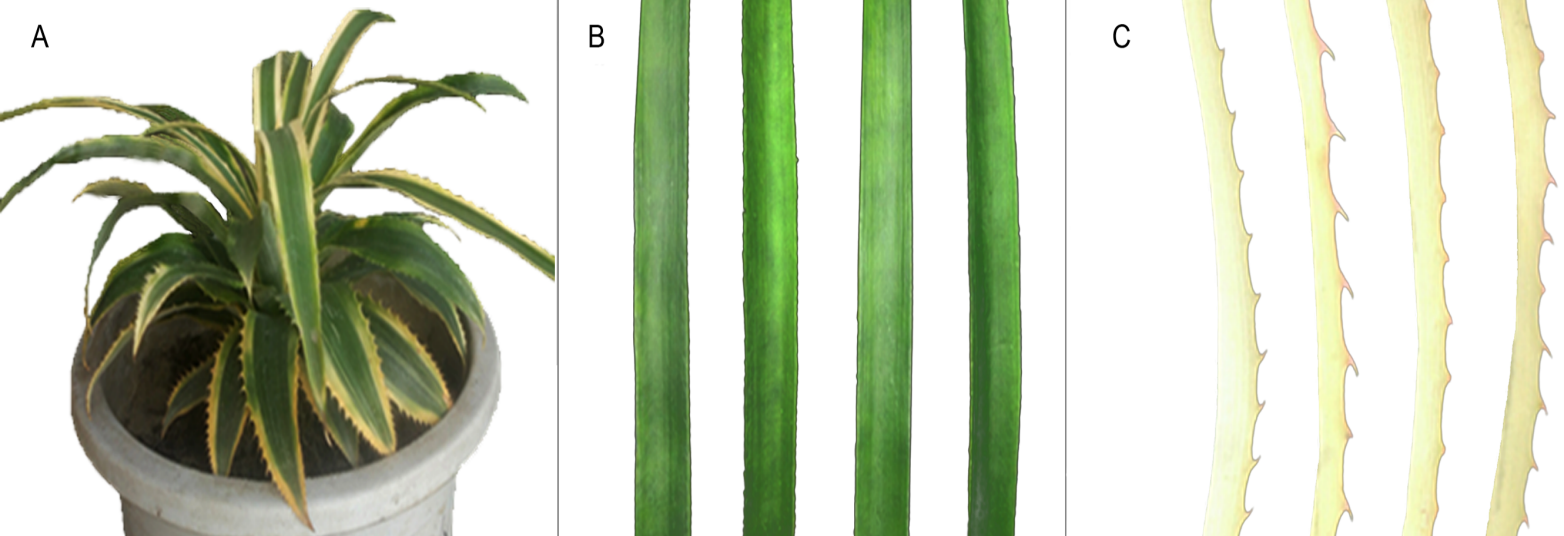
Phenotype of** chimeric leaves of * A. comosus* var. *bracteatus* used in this study. (A) Chimeric *A. comosus* var. *bracteatus*. (B) Green leaf strip from a chimeric plant. (C) White leaf strip from a chimeric plant.

Three-year-old *A. comosus* var. *bracteatus* chimeric plants were planted in the experimental field of Sichuan Agricultural University (Fig. 1). White parts and green parts of chimeric leaves were collected for transcriptomic sequencing and quantitative analysis of proteins. Three biological replicates of each sample were included and were obtained from ten plants. The samples were flash-frozen with liquid nitrogen and then stored at −80 °C for RNA-Seq and TMT analyses. The experimental system is depicted in Fig. S1.

### Contents of chlorophyll and carotenoid analysis

The contents of chlorophyll (Chl) and carotenoid (carot) in the white parts and green parts of *A*. *comosus* var. *bracteatus* were assessed. Chl a, Chl b, and carot levels were measured using the Holm equation and a method previously described (Holm, 1954).

### RNA extraction and RNA-Seq

Total RNA from the white and green parts of chimeric leaves was extracted separately using an RNeasy Plus Micro Kit (Qiagen, Hilden, Germany) following the recommendations of the manufacturer. The RNA integrity was assessed using RNA Nano 6000 Assay Kit and the Agilent 2100 Bioanalyzer system (Agilent Technologies, Santa Clara, CA, USA). Each RNA sample was subjected to DNase digestion (Takara) to remove any remaining DNA. mRNA enrichment, fragment interruption, adapter addition, size selection, PCR amplification and RNA-Seq were performed using the HiSeq X Ten platform (Illumina, San Diego, CA, USA).

### Protein extraction, digestion, and TMT labeling

Proteins were extracted using a previously published protocol, and the resulting proteins were digested by trypsin and TMT labeled as previously described (Guo et al., 2017). After trypsin digestion, peptides were desalted using a Strata X C18 SPE column.

### LC–MS/MS analysis

Peptide separation was performed using a reversed-phase analytical column (Acclaim PepMap RSLC; Thermo Scientific); peptides were subjected to a nanospray ionization source followed by MS/MS in Q Exactive™ PLUS (Thermo) coupled online to a UPL chromatograph. Peptides were selected for MS/MS using a normalized collision energy setting of 30, and ion fragments were detected in the Orbitrap at a resolution of 17,500. A data-dependent procedure that alternated between one MS scan and 20 MS/MS scans with a dynamic exclusion of 30.0 s was applied. For the MS scans, the m/z scan range was 350 to 1800, and the first fixed mass was set to 100 m/z.

### Database search

The resulting MS/MS data were processed using a MaxQuant search engine (v.1.5.2.8). Tandem mass spectra were searched against the database obtained from the client (27,024 sequences). Trypsin/P was specified as a cleavage enzyme with up to two missing cleavages. The mass error was set to 10 ppm for precursor ions and 0.02 Da for fragment ions. Carbamidomethyl on Cys was specified as a fixed modification, and oxidation on Met was specified as a variable modification. For protein quantification, TMT-6plex was selected in the MaxQuant search. The FDR was adjusted to <1%, and the peptide ion score was set to ≥20.

### Bioinformatics analysis

To identify the DEGs, all unigenes were searched for in the NCBI non-redundant (ncbi-nr) database as well as the Swiss-Prot database, eggNOG database and InterPro database. Analysis and identification of DEGs between Whs and Grs were performed using  R with edgeR package. Unigenes with an adjusted *p*-value <0.05 and |log2fold change| >1 between Whs and Grs were designated DEGs. Functional annotation of differentially accumulated transcripts and protein species were conducted using GO annotation and the KEGG database (Conesa et al., 2005; Tatusov et al., 2000). For protein quantitative analysis, a fold-change cut-off of >1.3 and <0.77 were set to identify protein species with high and low abundance, respectively, at a *p*-value <0.05.

## Results & Discussion

### Chlorophyll and carotenoid content analysis of Whs and Grs

Leaves of albino mutants have been associated with significantly lower chlorophyll and carotenoid contents than wild-type leaves (Liu et al., 2016a; Liu et al., 2016b). In our study, Chl and carotenoid contents were significantly different between Whs and Grs (Table 1), with Grs having approximately 16 and 17 times that in Whs, respectively. Chl a decreased significantly in Whs, accounting for only 3.21% of that in Grs, and Chl b in Whs accounted for 14.94% of that in Grs. Chl a/b levels were significantly lower in Whs than in Grs by 80%; thus, Chl a decreased more than did Chl b.

**Table 1:** Chlorophyll and carotenoid content in Green and White parts of chimeric leaves.

Sample	Total chl (mg g^−1^)	Chl a (mg g^−1^)	Chl b (mg g^−1^)	Chl a/b (mg g^−1^)	Carotenoid (mg g^−1^)
Green parts	0.621 ± 0.067a	0.467 ± 0.051a	0.154 ± 0.016a	3.038 ± 0.010a	0.0907 ± 0.0077a
White parts	0.038 ± 0.001b	0.015 ± 0.001b	0.023 ± 0.002b	0.638 ± 0.125b	0.0055 ± 0.0011b

**Notes.**

Note: Different letters within the same column indicate statistically significant differences (*P* < 0.05) according to a *T*-test.

### Transcriptomic analysis

#### Unigene functional annotation and DEG classification

Unigene sequences were queried via BLAST against the following databases: ncbi-nr database, the SwissProt database, the eggNOG database, the GO database and the KEGG database. Expression analysis and identification of DEGs between Whs and Grs were performed using the DEGseq R package (Wang et al., 2010). A *p*-value < 0.05 and a |log_2_ fold change (FC) | > 1 were set as criteria for the identification of DEGs, and 1,685 DEGs were identified. Among them, 973 and 712 transcripts were significantly down- and upregulated, respectively, which differs from the comparison of CGr and CWh generated via tissue culture, whereby more than twice as many genes were upregulated than were downregulated. Transcription levels in CWh were enhanced due to a compensatory mechanism caused by the lack of chlorophyll (Li et al., 2017), possibly because a plant with completely white leaves needs to activate metabolism to survive under the condition of a lack of chlorophyll. However, the white parts of chimeric leaves can collaborate with the green parts of chimeric leaves by each performing its own functions.

**Figure 2:**
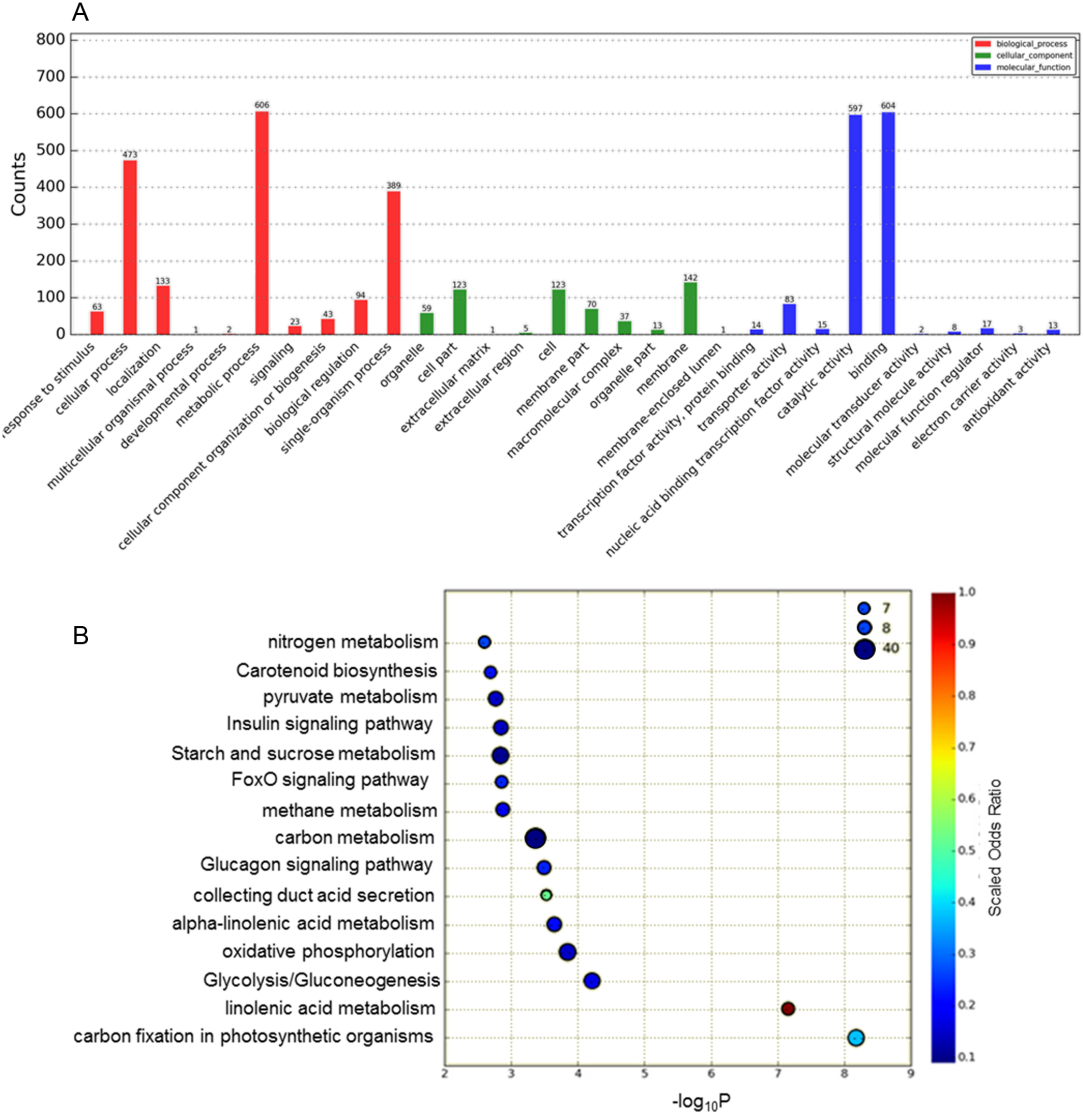
Analysis of DEGs based on the GO and KEGG pathways. (A) GO classification analysis of DEGs: (red) biological process, (green) cellular component, and (blue) molecular function. (B) The top 15 DEG-enriched KEGG pathways. Odds ratio: the greater the count ratio is, the greater the enrichment. −log_10_^*p*^: indicates the significance level after hypergeometric tests; a smaller value indicates a greater enrichment.

The functions of the DEGs obtained were classified according to the GO database Fig. 2A). In total, 1,685 DEGs were divided into the three categories of cellular component, molecular function and biological process, among which the majority of GO terms were assigned to biological process (48.6%), followed by molecular function (35.8%) and cellular component (15.2%). For the GO cellular component entries, DEGs were concentrated mainly in membranes, cells, cell parts and other components. The entries classified as having significant molecular function mainly included DEGs related to catalytic activity and binding. The biological process category was further divided into sub-categories, including metabolic process (606 genes), cellular process (473 genes), single-organismal process (389 genes) and other processes.

To further investigate the functions of these DEGs, statistical pathway enrichment analysis was performed based on the KEGG database, which mapped the DEGs onto 193 pathways. An overview of the functional cluster of the top 15 enriched pathways is shown in Fig. 2B. The significantly enriched pathways are related to intracellular energy metabolism processes (carbon fixation in photosynthetic, linoleic acid metabolism, glycolysis/gluconeogenesis) associated with carbohydrate and sugar metabolism, indicating differences in cell viability between Whs and Grs. Compared with previous research on plants with CGr and CWh, the pathways revealed in the present study are mainly involved in metabolism, especially in primary metabolism, whereas the enriched pathways between CGr and CWh were associated with photosynthesis, chlorophyll synthesis and carotenoid synthesis, i.e., mainly related to pigment biosynthesis. These findings indicate that the albino white cells in CWh are typical of the white cells of chimeric leaves.

#### Analysis of DEGs related to photosynthetic pigment and chloroplast development

A total of 11 unigenes related to chlorophyll biosynthesis were found based on KEGG pathway assignment, and all were upregulated in Whs. Compared to the transcriptome results of CGr and CWh, most of the DEGs in this study have similar mRNA expression levels, except for the *POR* gene. *POR* was the only gene detected to be downregulated in CWh but upregulated in Whs (Li et al., 2017). In addition, two unigenes associated with chlorophyll degradation were revealed by our KEGG pathway enrichment analysis. Chlorophyllase (Chlase) catalyzes the hydrolysis of chlorophyll into chlorophyllides and phytol, and this process is thought to be the initial step of the Chl-degradation pathway (Holden, 1961). In higher plants, NYC1 is a chlorophyll b reductase enzyme that catalyzes the first step of the conversion of chlorophyll b to chlorophyll a (Horie et al., 2009). The expression levels of *Chlase* and *NYC1* genes in CWh were enhanced (Li et al., 2017), and the similar results indicated that chlorophyll degradation may be activated both in Whs and CWh.

Carotenoids are essential components of the photosynthetic apparatus and photoprotection system (Demmig-Adams & Adams III, 1996). In the present work, the expression levels of the genes encoding violaxanthin de-epoxidase (*VDE*), phytoene synthase (*PSY*) and phytoene desaturase (*PDS*) were higher in the white parts than in the green parts of chimeric leaves. Analysis of physiological indicators showed that the carot content in white parts was very low, accounting for 1% of that in green parts. Carotenogenic genes are nuclearly encoded, but the enzymes participate in the biosynthetic pathway in the plastid. According to the plastid signal hypothesis, expression of nuclear-encoded plastid enzymes is regulated by signals from the plastid (Wu, 2015). In the present study, high expression of *PSY, PSD* and *VDE* were found in white tissues with abnormal chloroplast development, indicating that maintaining high expression of carotenoid-related enzyme genes is not completely dependent on normal chloroplasts but is negatively affected by plastid signals. Furthermore, the xanthophyll cycle within the carotenoid pathway plays an important role in protection under environmental stress. VDE catalyzes the conversion violaxanthin to zeaxanthin, a compound with photoprotective function. The *VDE* gene is more highly expressed under adverse conditions, and *VDE* was upregulated in our transcriptome in Whs.

Many albino mutants have been reported to be impaired in chloroplast differentiation and development (Nien et al., 2007). The *Golden2-like (GLK*) gene family regulates chloroplast development in both corn (Liu et al., 2016a; Liu et al., 2016b) and *Arabidopsis thaliana* (Fitter et al., 2002). Additionally, the *Ftsz* gene family has been shown to be essential for chloroplast division in higher plants. In the present study, compared with Grs, the expression level of *GLK1* in Whs was downregulated, and the *FtsZ* gene was upregulated in Whs/Grs. Accordingly, *GLK* and *FtsZ* may regulate chloroplast development and division.

#### Analysis of DEGs in Nitrogen metabolism

Ammonia is assimilated in plants via the glutamine synthetase/glutamate synthase (GS/GOGAT) cycle or the glutamate dehydrogenase (GDH) pathway (Miflin & Habash, 2002). The former is the major pathway for ammonia assimilation in higher plants, whereas the key enzyme GS plays a central role in nitrogen metabolism in plants. It has been reported that *GS* overexpression can improve the efficiency of nitrogen use and promote growth (Cai et al., 2009). In addition, the GDH pathway supplements the GS/GOGAT cycle for ammonia assimilation. The results of our transcriptomics analysis showed that the expression levels of *GS* and *GDH* were lower in white parts compared to green parts, indicating that Whs had a weaker ability to assimilate nitrate.

### Proteomic analysis

#### Identification and functional classification of DAPs

As protein abundance levels cannot always be accurately predicted based on quantitative mRNA data, a comparison of the proteome between Whs and Grs was performed. In total, 31,808 peptides, 29,262 unique peptides and 5,428 protein species were identified (Table 2). The peptide sequences and charges are presented in Table S1. Among the protein species, 4,791 were quantified, and among the quantified protein species, 1,018 exhibited high abundance and 795 low abundance in Whs. The number of DAPs was slightly greater than the number of DEGs. Overall, the abundance of protein species does not well agree with the expression levels of the encoding genes because posttranscriptional regulation affects the abundance of protein species (Yan et al., 2006). Identification of such protein species will contribute greatly to our understanding of the mechanism responsible for the albino parts of the chimeric leaves and the growth and development of chimeric plants.

**Table 2:** Peptides and protein species identified of MS/MS database search.

Peptides	Unique peptides	Identified proteins	Quantifiable proteins	DAPs
31808	29262	5428	4791	1813

**Figure 3:**
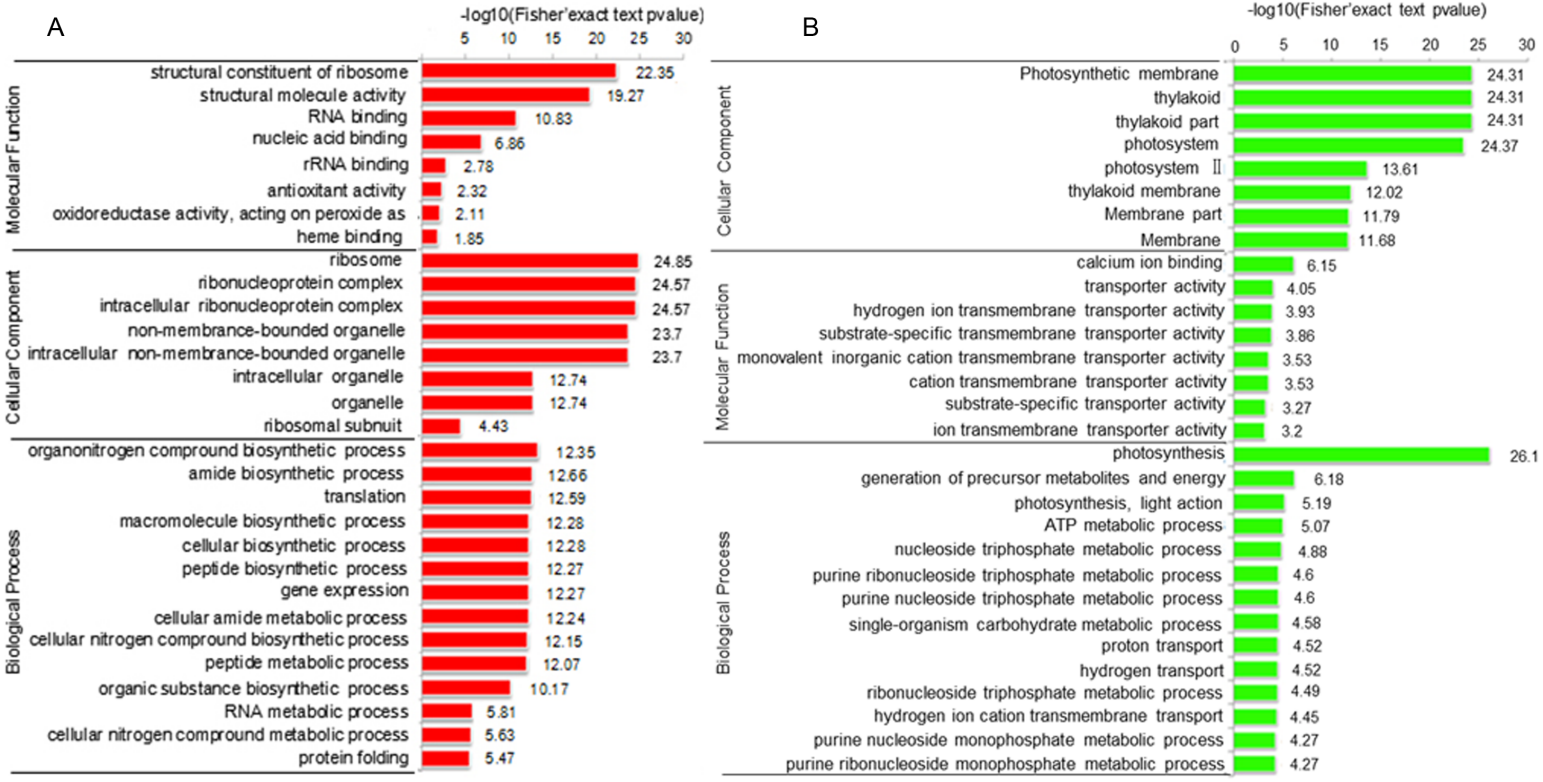
Functional categorization of DAPs between Whs and Grs based on GO annotations. (A) GO-based enrichment analysis of up-accumulated protein species. (B) GO-based enrichment analysis of down-accumulated protein species.

To elucidate the function of DAPs based on GO enrichment, the quantified protein species were analyzed via cluster analysis (Fig. 3A). We found that DAPs were mostly enriched in the cellular component category, followed by the biological process and molecular function categories. In cellular component, the highly abundant protein species were enriched in categories of ribosomes and ribonucleoprotein complex, whereas those with low abundance were enriched in photosynthetic membranes, thylakoids, and photosystems. Previous studies have shown that leaf color and photosynthetic efficiency are directly affected by chloroplast development, the number and size of chloroplasts, and chlorophyll biosynthesis and content (Zhang et al., 2006; Ma et al., 2016; Yang et al., 2015). Our results indicated that the abundance of protein species related to photosynthesis was significantly low in Whs (Fig. 3B). In terms of biological processes, abundant protein species were highly enriched in the organonitrogen compound biosynthetic process, translation, macromolecule biosynthetic process and others. Analysis of molecular functions revealed that many of the abundant protein species were highly enriched in categories of structural constituents of ribosomes, structural molecule activity, and RNA binding.

**Figure 4:**
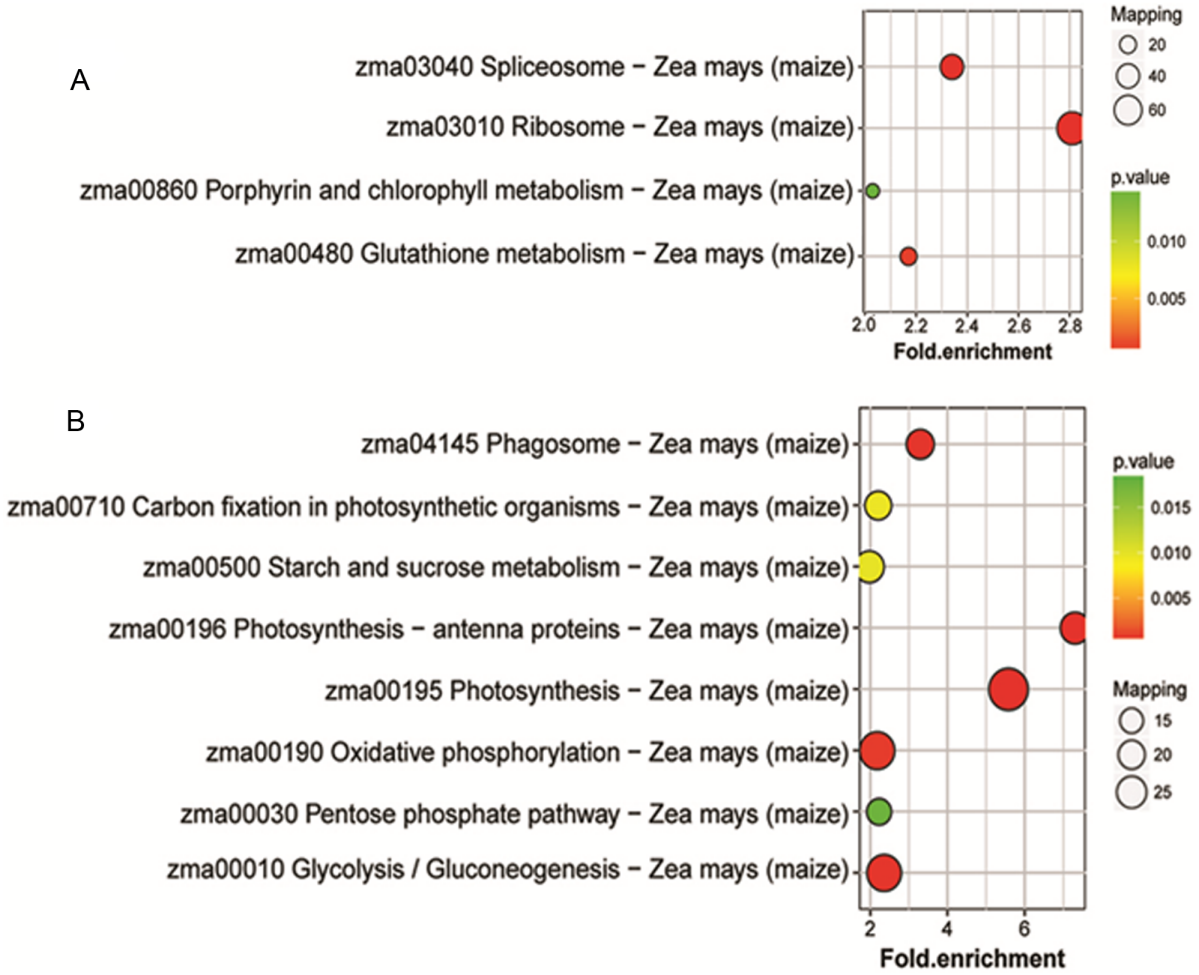
Enriched pathways of protein species between Whs and Grs of chimeric leaves as determined by KEGG analyses. (A) Up-accumulated protein pathway enrichment. (B) Down-accumulated protein pathway enrichment.

To identify the metabolic pathways that might be involved in albinism, all DAPs were queried against the KEGG database, which resulted in their mapping to eight main KEGG pathways. The highly abundant protein species, as determined by comparison of Whs to Grs, are primarily related to ribosomes, spliceosomes, glutathione metabolism and porphyrin and chlorophyll metabolism. An overview of the DAP-enriched KEGG pathways is illustrated in Figs. 4A, 4B.

#### Analysis of DAPs related to chlorophyll biosynthesis, photosynthesis antennas and photosynthesis

A total of 18 DAPs in Whs, including six with low abundance and 12 with high abundance, were identified in the porphyrin and chlorophyll metabolism pathway. Notably, the abundance of magnesium-protoporphyrin IX methyltransferase (CHLM) and divinyl chlorophyllide a 8-vinyl-reductase (DVR), the rate-limiting enzymes of chlorophyll synthesis, were significantly lower in Whs than in Grs. These findings suggest that the albino phenotype of Whs might be caused by a low abundance of key enzymes involved in the chlorophyll biosynthesis pathway. In addition, the reduced abundance of NYC1, which controls the transformation of Chl b to Chl a likely led to the observed Chl a/b decrease in Whs.

**Figure 5:**
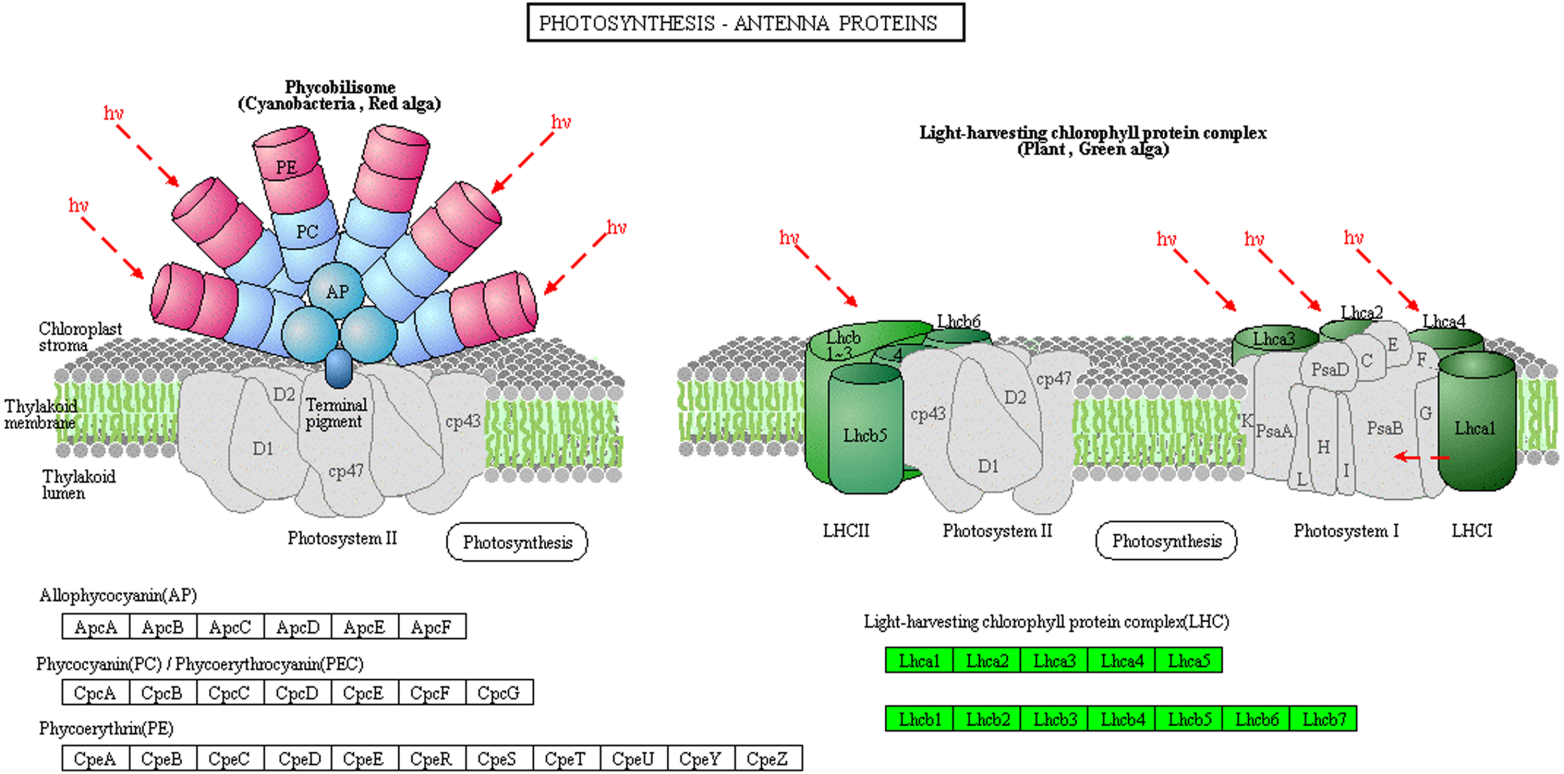
Photosynthesis-antenna protein pathways obtained from KEGG pathway analyses. The protein species in green are down-accumulated.

The light-harvesting chlorophyll protein complex (LHC), which is located on the periphery of the photosystems, plays an important role in light harvesting in PSI and PSII. Low levels of LHC proteins result in abnormal chloroplast development (Kim et al., 2009; Li, 2015). In this study, the abundance of chlorophyll complex protein I (Lhca1-5) and chlorophyll complex protein II (Lhcb1-7) was lower in Whs compared with Grs (Fig. 5). The lack of LHC function may affect the capture and transmission of light energy in the chloroplast.

**Figure 6:**
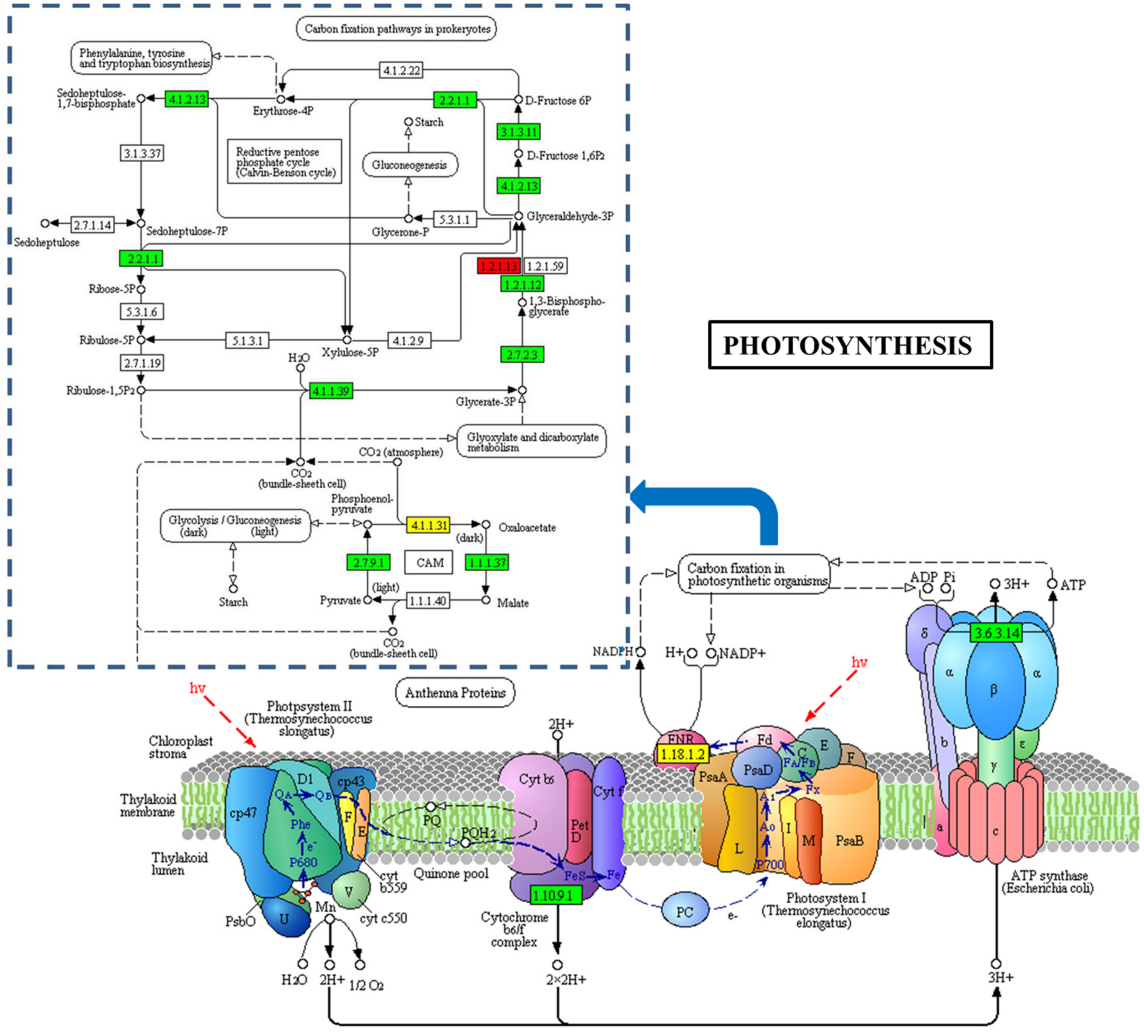
DAPs involved in photosynthesis between the Whs and Grs. The blue dashed wireframe outline indicates the protein species that are involved in carbon fixation in photosynthetic organisms.

Additionally, 13 DAPs are involved in carbon fixation in photosynthetic organisms. Of these, 10 were reduced in abundance, including ribulose-1,5-bisphosphate carboxylase oxygenase (Rubisco), NADP-malate dehydrogenase (NADP-MDH), phosphoglycerate kinase (PGK), and glyceraldehyde 3-phosphate (GAPDH) (Fig. 6). Rubisco is a key enzyme in photosynthesis and catalyzes the first step of the Calvin cycle for carbon assimilation (Spreitzer & Salvucci, 2002). PGK is a monomeric enzyme that transfers the high-energy phosphate group of 1,3-bisphosphoglycerate to ADP to form ATP and 3-phosphoglycerate (Li et al., 2011). In C4 plants, NADP-MDH functions to reduce oxaloacetate (OAA), the product of primary CO_2_ fixation by phosphoenolpyruvate carboxylase (PEPC), to malate; the malate produced is then transported to adjacent bundle sheath cells (Cushman, 1993). *Ananas* carries out crassulacean acid metabolism (CAM), a related process (Li et al., 2017). The low abundance of these protein species indicates that the CO_2_ fixation capacity of Whs differs from that of Grs, which may slow the photosynthesis process in Whs.

#### Analysis of DAPs in Ribosome

A total of 76 DAPs in the ribosome metabolic pathway were identified based on KEGG pathway assignments: 70 of these protein species were increased in abundance and the other six decreased. The abundance of the ribosomal large subunit proteins L11, L3e, L19e, and L33 and the ribosomal small subunit protein S18e (RSP18) were significantly diminished in Whs compared to Grs. Previous studies have shown that the loss of *rps18*, a chloroplast ribosomal small subunit-encoding gene, in tobacco results in the formation of an albino phenotype in leaves and their eventual death (Hörtensteiner, 2006). Moreover, the levels of multiple large and small subunit genes encoding ribosomal proteins were significantly altered in *Oncidium* ‘Milliongolds’, leading to a chlorotic phenotype (Tian, 2016). Thus, the low abundance of these protein species may play a role in leaf albinism.

#### Transcriptomic and proteomic cross-talk analysis

**Figure 7:**
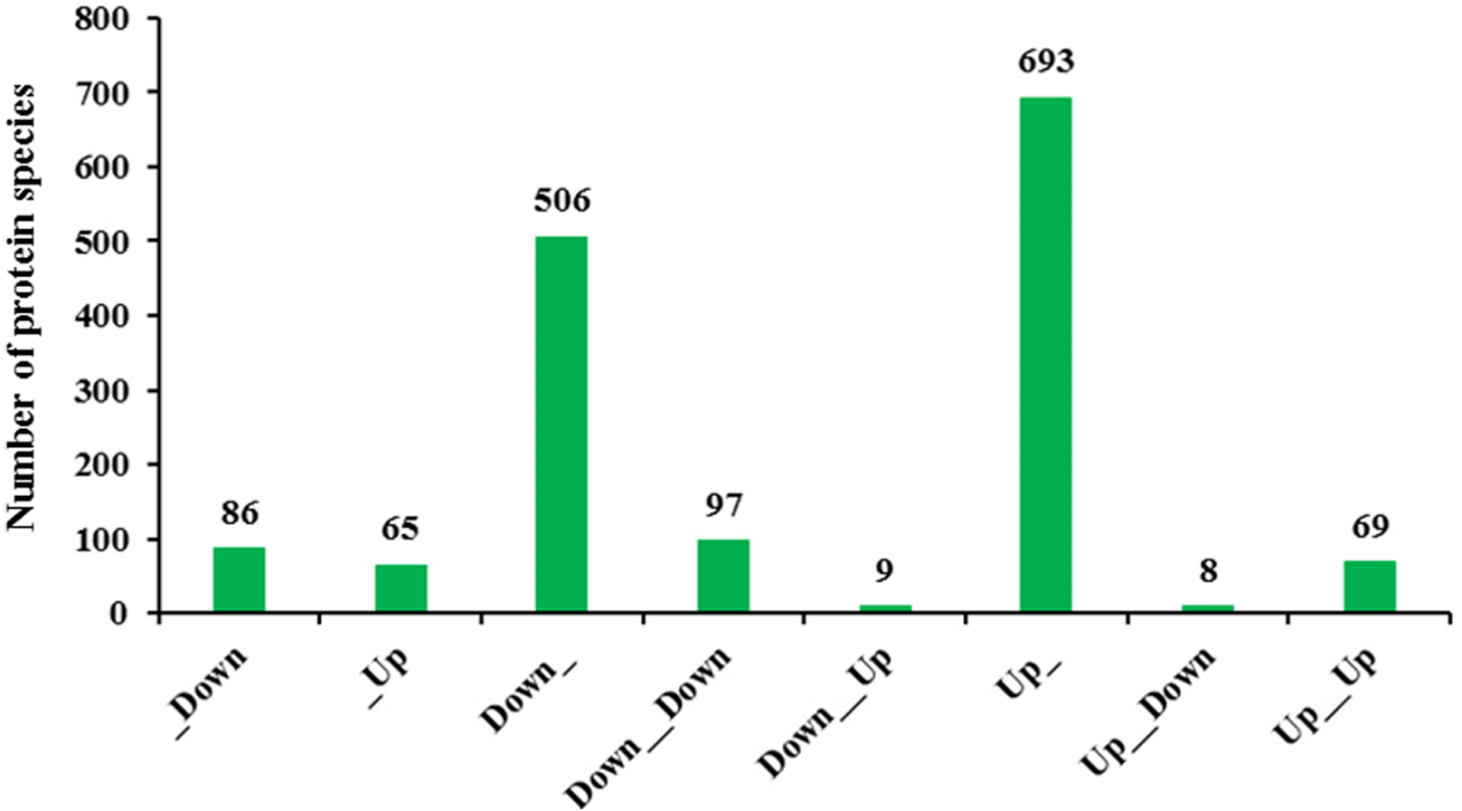
Transcriptomic and proteomic quantification relationships. _Down: Protein unchanged, transcription down-accumulated; _Up: Protein unchanged, transcription up-accumulated; Down_: Protein down-accumulated, transcription unchanged; Down_Down: Protein and transcription down-accumulated; Down_Up: Protein down-accumulated, transcription up-accumulated; Up_: Protein up-accumulated, transcription unchanged; Up_Down: Protein up-accumulated, transcription down- accumulated;Up_Up:Protein up-accumulated, transcription up-accumulated.

**Figure 8:**
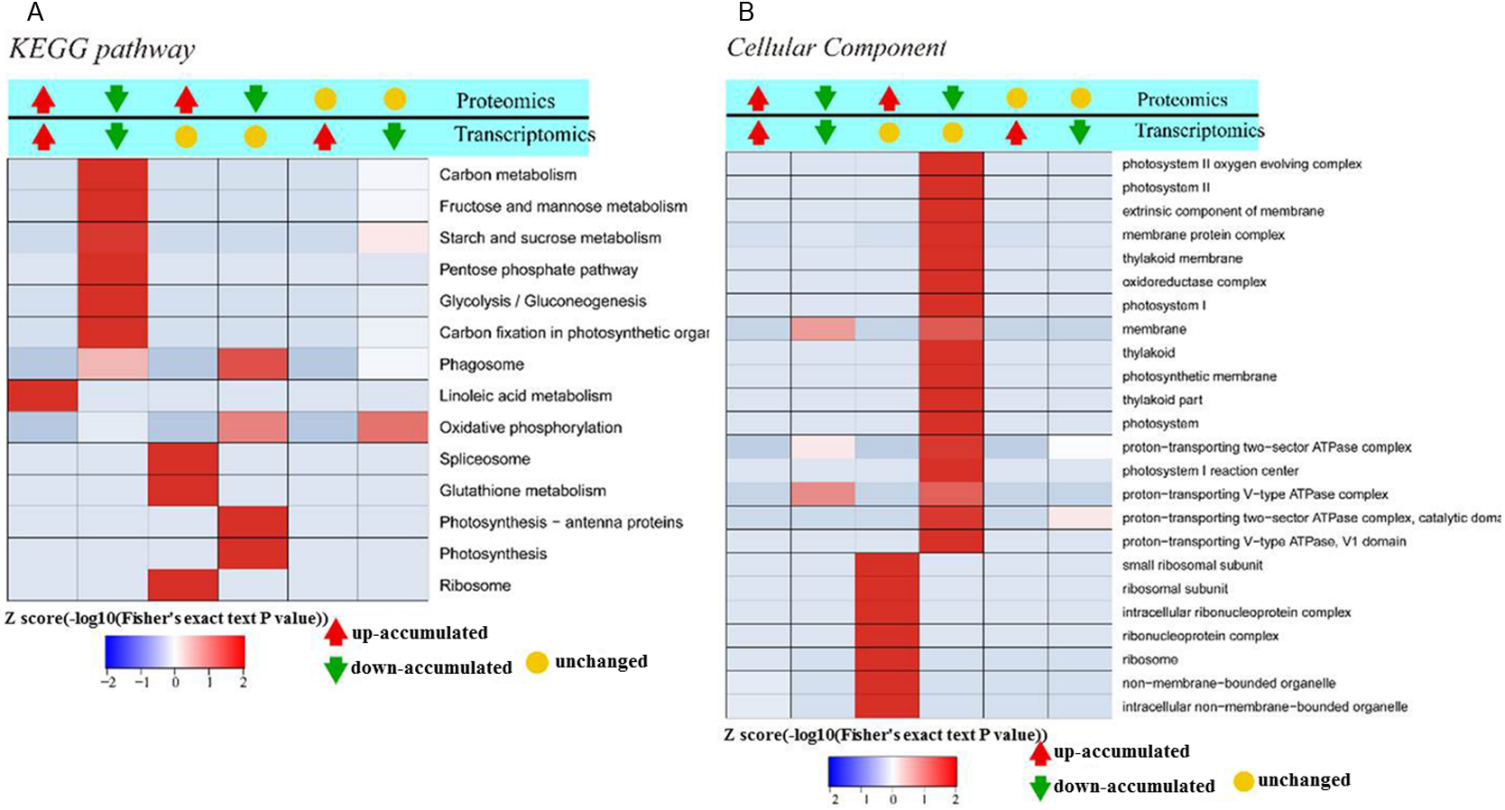
Significant enrichment analysis of DEGs with respect to protein and mRNA based on the direction of change. (A) Significantly enriched DEGs involved in different KEGG pathways. (B) Significantly enriched DEGs classified as belonging to different cellular components.

Transcriptomic analysis enables a more in-depth understanding of gene expression networks, and proteomics links these networks to protein products and provides deeper insight into posttranscriptional modifications (Wang et al., 2014). As gene transcription data are insufficient for predicting protein abundance (Wang et al., 2016), evaluation of the crosstalk relationship between proteome and transcriptome was conducted. Our comparative analysis results showed that protein abundances were not positively related with transcription levels. In fact, only 166 protein species exhibited accumulation patterns consistent with those of their mRNAs (Fig. 7). Among them, 69 protein species and their coding mRNAs were both significantly increased and 97 protein species and their coding mRNAs were both decreased in Whs compared with Grs. The up_up protein species were enriched in linoleic acid metabolism, whereas the down_down protein species were enriched in carbon metabolism pathways, such as carbon fixation in photosynthetic, starch and mannose metabolism (Fig. 8A). In addition, 693 protein species were increased at the proteomic level but unchanged at the mRNA level, and 506 protein species were decreased at the proteomic level but unchanged at the mRNA level. Numerous reports have suggested that RNA transcript accumulation does not always agree with the final protein product, as the steps of transcription of DNA into mRNA and translation of mRNA into protein are regulated by various factors during the processes of transcription, translation and posttranslational regulation (Deribe, Pawson & Dikic, 2010; Shemesh-Mayer et al., 2015; Shi et al., 2017). In our study, GO analysis showed that low-abundance protein species with unchanged mRNA expression levels were enriched in photosystem, thylakoid, and membrane components (Fig. 8B). Notably, nine protein species with reduced abundance but with upregulated gene expression were identified in Whs (Table 3).

**Table 3:** Up_Down quantification relationship of transcriptomics and proteomics.

		Transcriptomics				proteomics		
Protein accession	Protein description	logFC (Wh/Gr)	*p*-value	*q*-value	Regulated Type	Wh/Gr Ratio	Wh/Gr *P* value	Regulated type
Aco000471.1	Putative L-ascorbate peroxidase 6	3.976	0.00047367	0.01422146	_Up	0.473	0.0000161	Down_
Aco005951.1	”Chlorophyll a-b binding protein	2.538	9.3709E−11	2.2658E−08	_Up	0.096	0.0121	Down_
Aco006504.1	”Preprotein translocase subunit SECY	1.038	9.4357E−05	0.00405446	_Up	0.404	0.0000372	Down_
Aco008453.1	Chloroplastic lipocalin	1.198	0.00026127	0.00899766	_Up	0.521	0.000000311	Down_
Aco013684.1	”PsbP domain-containing protein 1	1.368	7.7316E−05	0.0034582	_Up	0.236	0.0000624	Down_
Aco018356.1	”Divinyl chlorophyllide a 8-vinyl-reductase	1.156	0.00013306	0.00531348	_Up	0.317	0.00000147	Down_
Aco021970.1	”Photosynthetic NDH subunit of lumenal location 3	1.580	0.00097191	0.02464119	_Up	0.162	0.00000133	Down_
Aco023399.1	”Rhodanese-like domain-containing protein 14	1.157	0.00193652	0.04114251	_Up	0.479	0.000103	Down_
Aco024665.1	”Lycopene beta cyclase	1.355	2.2429E−05	0.00126599	_Up	0.714	0.0279	Down_

**Notes.**

Protein accession numbers can be found at https://phytozome.jgi.doe.gov/pz/portal.html.

Chlorophyll and carotenoids are the principal pigments that trap light energy, and as the main component of the light-harvesting complex, chlorophyll plays an important role in photosynthesis (Sandhu et al., 2016). Moreover, altered chlorophyll metabolism is one of the most important factors leading to the albino phenotype. CHLM and DVR are key enzymes involved in the synthesis of chlorophyll, and RuBPCase, NADP-MDH, cp43, cp47, LHC and PsbP are key enzymes related to photosynthesis; LCYB is crucial for the synthesis of carotenoids. However, the expression levels of the genes coding for these enzymes did not well agree with the abundance of the protein species, especially for DVR, CHLM, LCYB, LHC and PsbP. In addition, the abundance of DVR, CHLM, LCYB, LHC and PsbP proteins was reduced in Whs while the expression levels of their genes was upregulated in Whs. These results suggest that posttranscriptional regulation, non-coding RNAs, alternative splicing, and posttranslational modifications may play important roles in the albino phenotype of chimeric leaves.

## Conclusion

In this study, global and comparative transcriptomic and proteomic analyses were performed to compare the white and green parts of the chimeric leaves of *A. comosus* var. *bracteatus*. Combined analyses of the transcriptome and proteome revealed that the abundance of protein species was not well in accordance with the expression levels of the encoding genes. Nine protein species with low abundance in Whs were identified, even though the gene expression levels were upregulated, including five genes related to chlorophyll biosynthesis, carotenoid biosynthesis and photosynthesis. These findings suggest that transcription, posttranscriptional modification and translation processes may play important roles in the albino phenotype of chimeric leaves.

##  Supplemental Information

10.7717/peerj.7261/supp-1 Figure S1 The experimental system of this study Three biological replicates of the white parts and green parts of chimeric leaves were used for RNA-Seq and LC-MS/MS analysis. And evaluation of the crosstalk relationship between proteome and transcriptome was conducted.

10.7717/peerj.7261/supp-2 Table S1 Identification and quantification of peptides by Mass spectrum The sheet of quantification of peptides. **Sequence**: Identified peptide amino acid sequence. **Charge:** Carried charge of peptide. **m/z**: Mass-to-charge ratio of peptide. **mass error [ppm]**: Parts per million ratio of peptide mass error between the theory and practice. **PEP**: The maximal posterior error probability for peptides. **Score**: A simple rule to be used to judge whether a result is significant or not. **Unique [yes/no]**: When marked with ’yes’, this particular peptide is unique to a single identified protein group.

10.7717/peerj.7261/supp-3 Table S2 Mass spectrum identified information Table of quantified proteins and the number of peptides whitch spetrums hit. **95%CI**: confidence interval set as 95%. **MW**: Protein molecular weight, unit [kDa]. **Coverage**: Percent of identified peptdie sequence covering in protein sequence. **Peptides**: Number of identified peptides. **Unique peptides**: Number of identified peptides that only come form this protein groups. **PSMs**: Number of spectrum which protein matched.

10.7717/peerj.7261/supp-4 Table S3 T-text of statistical analysis for the contents of chlorophyll and carotenoid by SPSS Two independent samples of t-text was used in this analysis.

## Additional Information and Declarations

### Data Availability

The following information was supplied regarding data availability: The raw data is available at ProteomeXchange, project accession: PXD010375.
