# Supplementary figures and images for: Comparative transcriptomic and proteomic analyses of the green and white parts of chimeric leaves in Ananas comosus var. bracteatus
Source: PeerJ. 2019 Jul 10;7:e7261. doi: 10.7717/peerj.7261 (PMC6626515; doi:10.7717/peerj.7261)

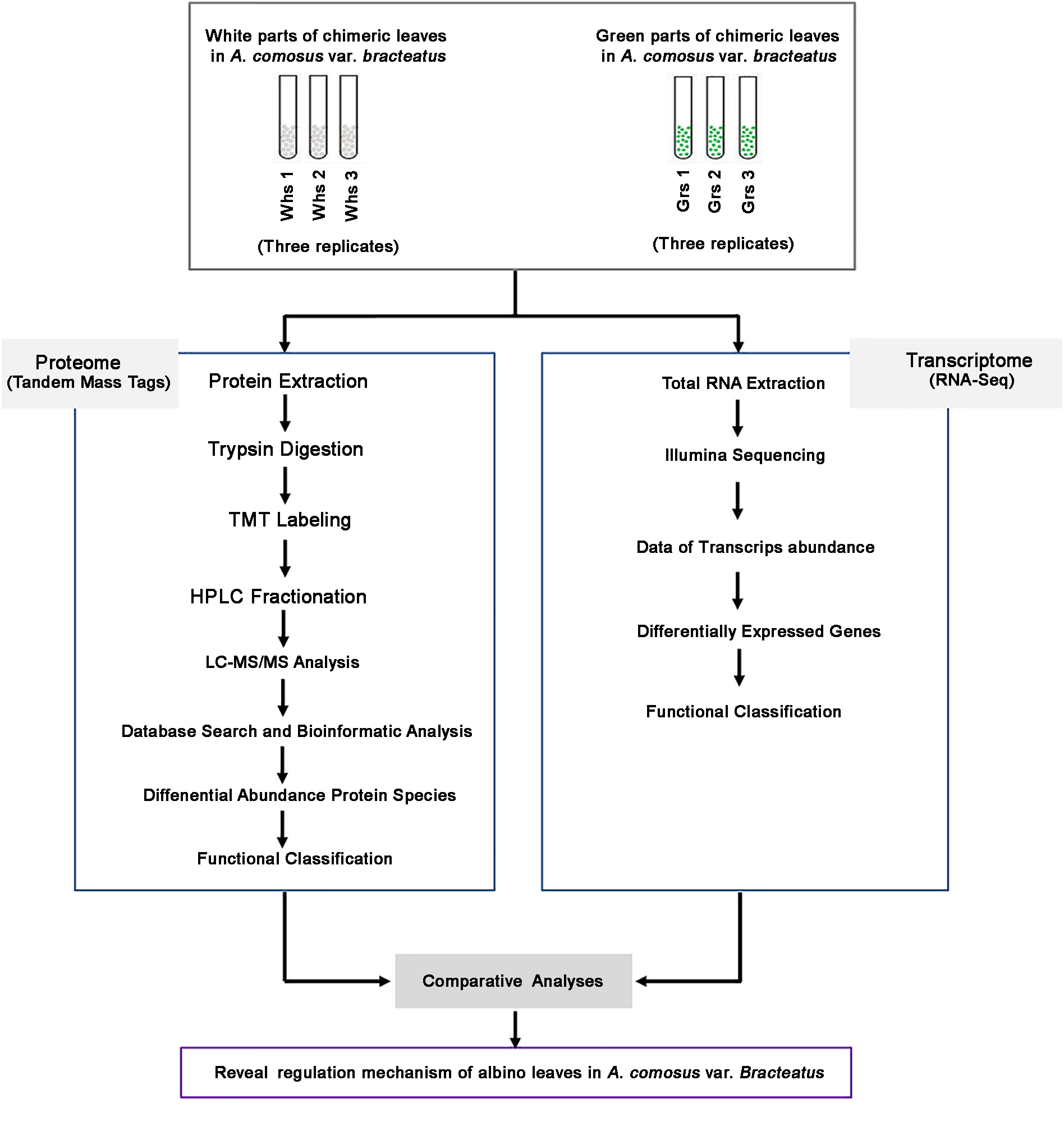

Supplement: Figure S1 — Three biological replicates of the white parts and green parts of chimeric leaves were used for RNA-Seq and LC-MS/MS analysis. And evaluation of the crosstalk relationship between proteome and transcriptome was conducted. [file peerj-07-7261-s001.png]

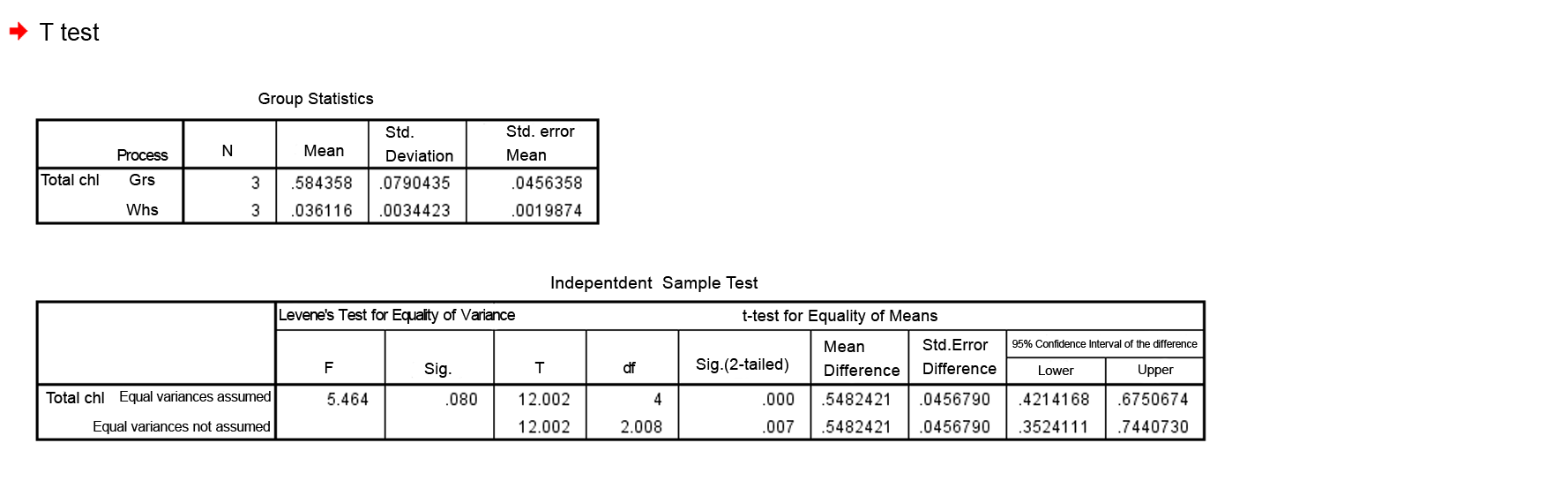

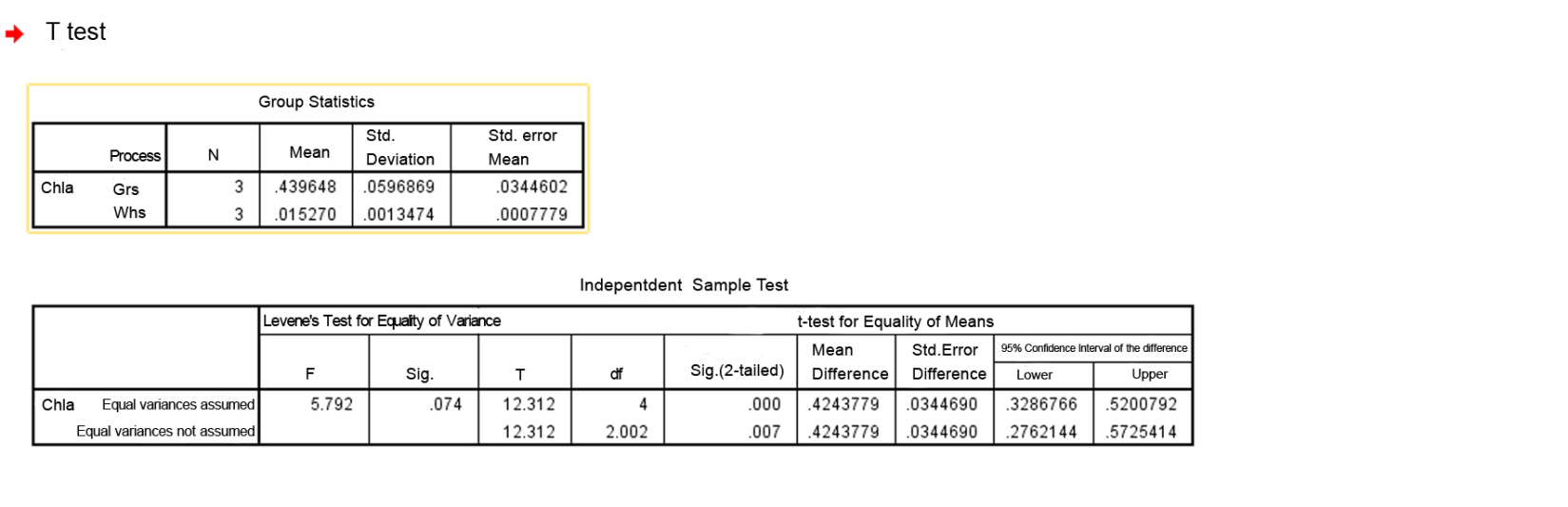

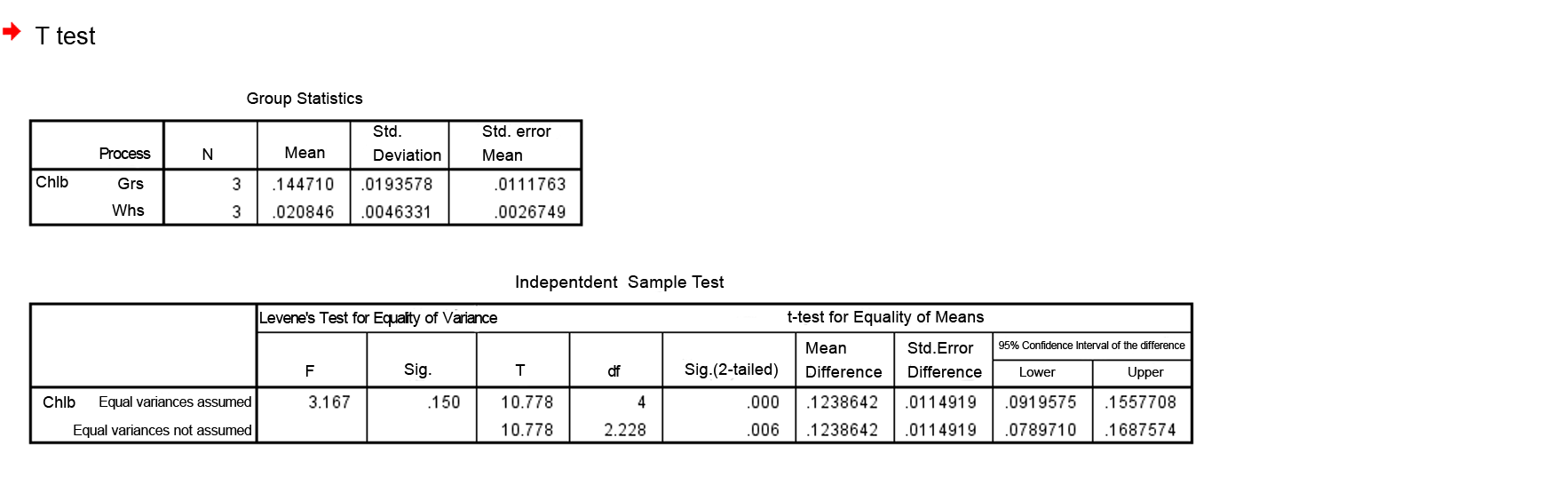

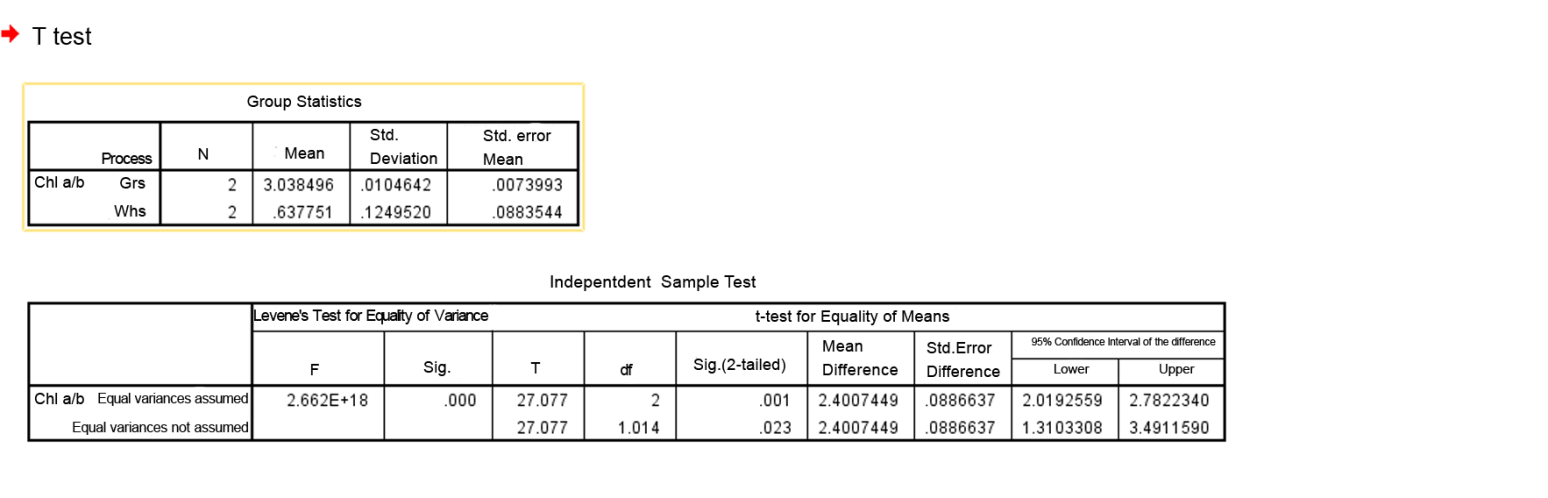

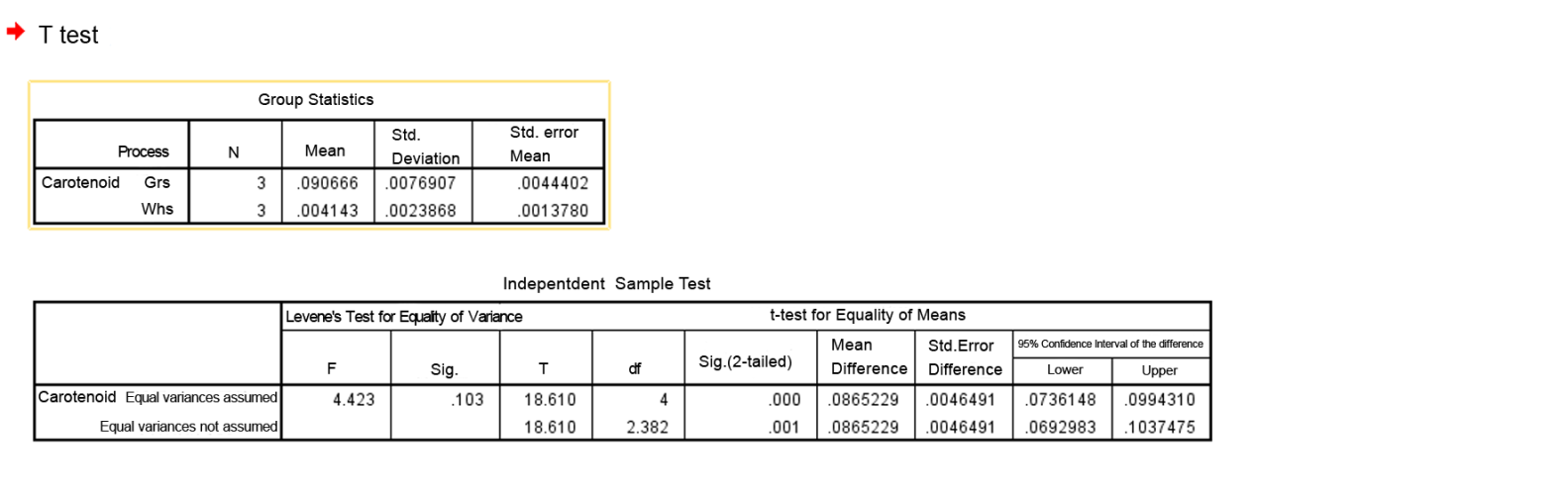

Supplement: Table S3 — Two independent samples of t-text was used in this analysis. [file peerj-07-7261-s004.docx]
